# Location Privacy Protection for the Internet of Things with Edge Computing Based on Clustering K-Anonymity

**DOI:** 10.3390/s24186153

**Published:** 2024-09-23

**Authors:** Nanlan Jiang, Yinan Zhai, Yujun Wang, Xuesong Yin, Sai Yang, Pingping Xu

**Affiliations:** 1 School of Communication and Artificial Intelligence, School of Integrated Circuits, Nanjing Institute of Technology, Nanjing 211167, China; 2 Electrical and Computer Engineering Faculty, Brown University, Providence, RI 02903, USA; 3 National Mobile Communications Research Laboratory, Southeast University, Nanjing 211189, China

**Keywords:** Internet of Things, edge computing, location privacy, K-anonymity, cluster

## Abstract

With the development of the Internet of Things (IoT) and edge computing, more and more devices, such as sensor nodes and intelligent automated guided vehicles (AGVs), can serve as edge devices to provide Location-Based Services (LBS) through the IoT. As the number of applications increases, there is an abundance of sensitive information in the communication process, pushing the focus of privacy protection towards the communication process and edge devices. The challenge lies in the fact that most traditional location privacy protection algorithms are not suited for the IoT with edge computing, as they primarily focus on the security of remote servers. To enhance the capability of location privacy protection, this paper proposes a novel K-anonymity algorithm based on clustering. This novel algorithm incorporates a scheme that flexibly combines real and virtual locations based on the requirements of applications. Simulation results demonstrate that the proposed algorithm significantly improves location privacy protection for the IoT with edge computing. When compared to traditional K-anonymity algorithms, the proposed algorithm further enhances the security of location privacy by expanding the potential region in which the real node may be located, thereby limiting the effectiveness of “narrow-region” attacks.

## 1. Introduction

The proliferation of cost-effective micro-devices with computational and communication capabilities is providing promising solutions to enhance the quality of life. These micro-devices perform well in a multitude of ubiquitous application areas, including, but not limited to, smart cities [1], meteorology [2], healthcare systems [3], smart grids [4], industrial automation, intelligent automated guided vehicles (AGVs), and precision agriculture [5]. With the development of the Mobile Ad Hoc Network (MANET) [6], the devices mentioned above, along with other devices, together constitute the Internet of Things (IoT) [7].

However, due to the interconnection of unattended and globally accessible devices with the untrusted and unreliable Internet, IoT technology is vulnerable to external attacks [8]. These system vulnerabilities motivate adversaries to launch different attacks, such as forgery and Sybil attacks [9], false data injection [10], replay attacks, and denial of service attacks.

In recent years, edge computing has become popular among the research community [11]. Compared to its predecessor, the cloud computing paradigm, it has advantages in protecting privacy, decreasing latency, and reducing network bandwidth, which are essential for providing safer services for each node in a transmission [12]. The integration of edge computing will definitely improve the efficiency of the IoT. For instance, the data collected or transmitted by nodes of the IoT, such as Wireless Sensor Networks (WSNs) [13], have a strong correlation with location information [14,15], and some of these data can be processed by an edge computing device, such as a coordinator, rather than the central node. Additionally, in a network that is constituted by high-speed nodes, such as a Vehicular Ad Hoc Network (VANET) [16], location information is also necessary for geographic routing [17], where the processing of related data can be achieved by an edge computing device, such as a Roadside Unit (RSU), rather than the control center [18]. In the cases above, attackers cannot listen to or falsify the location information of the nodes by attacking the remote server, thus improving location privacy. However, a system that operates in an open environment is easily exposed to eavesdroppers, which can result in the leaking of location information. Therefore, it is necessary to protect the location privacy of the IoT nodes at the edge computing devices and during the communication process.

To protect users’ privacy, many Location Privacy Protection Mechanisms (LPPMs) have been proposed [19]. The purpose of LPPMs is to protect users’ privacy effectively while allowing them to enjoy LBS [20]. Some location privacy protection algorithms can be applied to various scenarios, while others are designed specifically for certain scenarios. The advantages and disadvantages of the above algorithms depend on the application environment and users’ needs, such as the level of privacy protection.

K-anonymity is one of the typical LPPMs [21] and is easily applicable. K-anonymity can safeguard the location information of a group of nodes by incorporating fake information, which can be the locations of nodes outside the group or virtual locations. However, it remains unclear which type of locations, virtual or other real ones, should be utilized to protect location privacy. Furthermore, in the context of the IoT with edge computing, service providers are distributed, creating uncertainty for attackers in determining which edge computing device to target [22]. Additionally, some IoT nodes, particularly low-power-consumption devices, transmit their locations together without encryption to reduce communications consumption, rendering wireless links vulnerable to attackers [23]. Unfortunately, most LPPMs focus primarily on the security of data at the service provider, which is inadequate for IoT environments with edge computing capabilities [24].

To address the aforementioned research gaps, an improved K-anonymity algorithm, the Clustering K-anonymity Algorithm (CKA), is proposed in this paper. CKA can adapt to different environments due to the utilization of virtual locations. Additionally, in certain scenarios, eavesdroppers may only require a limited region of a user’s location; this type of attack is referred to as a “narrow-region” attack throughout the remainder of this paper. Traditional K-anonymity with real locations is unable to defend against narrow-region attacks [25], and the K-anonymity with virtual locations can only significantly reduce transmission efficiency. Therefore, a security estimation scheme based on the K-function is proposed to evaluate the performance of CKA in defending against a narrow-region attack.

CKA can enhance the location privacy security of the IoT by clustering nodes, and the location of each node is further obscured through the regional division, which can confuse eavesdroppers when they narrow the search region. Furthermore, compared to the traditional K-anonymity algorithm that relies solely on the locations of other nodes, the successful probability of information transmission from the nodes to the service provider is improved in CKA. Simulation results demonstrate that, under the same conditions, CKA provides significant security improvements over traditional algorithms across any density of nodes, where security improvement refers to the reduced probability of eavesdroppers revealing location information. Distinct from the traditional K-anonymity algorithm with real locations [26], CKA can effectively confound eavesdroppers as they attempt to narrow the search region according to the locations of nodes. The major contributions of this article are summarized as follows:

(1) A clustering idea is used to improve the location privacy security of IoT nodes with edge computing.

(2) The location of each node is further obscured through a regional division, which can confuse eavesdroppers when they narrow the search region.

(3) We improve the successful probability of information transmission by the head node compared to the traditional K-anonymity algorithm with false locations.

(4) A security estimation scheme for defending against narrow-region attacks based on the K-function is proposed to optimize the application of CKA.

The rest of the paper is organized as follows: Section 2 discusses related work on CKA. In Section 3, the system model of CKA is described. The details of CKA are introduced in Section 4. We evaluate the performance of CKA in Section 5. In Section 6, the data efficiency and the security estimation scheme for defending against a narrow-region attack are discussed. Finally, we conclude the paper in Section 7.

## 2. Related Work

Location privacy protection algorithms encompass a broad spectrum of technologies, ranging from data perturbation to data encryption and the generation of fake data. LPPMs play a pivotal role in protecting user privacy, and CKA itself has evolved from traditional LPPMs. Consequently, comprehending the essence of LPPMs is paramount. Furthermore, existing privacy protection algorithms can provide novel insights for devising protection strategies, thereby making an understanding of these algorithms equally essential for the development of CKA. The following subsections will elaborate on these two perspectives.

### 2.1. The Classification of LPPMs

**Figure 1:**
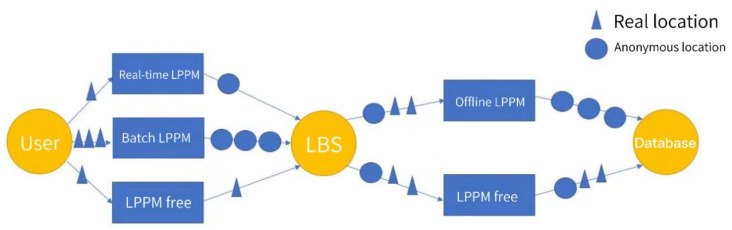
LPPM scenario classification.

LPPMs are essential for safeguarding users’ privacy while utilizing LBS. These mechanisms vary in their application, with some suited for general use and others designed for specific scenarios. The effectiveness of LPPMs depends on the environment and users’ privacy needs. There are two main classifications of LPPMs, online LPPMs, and offline LPPMs, as shown in Figure 1. In online LPPMs, there are real-time LPPMs and batch LPPMs to solve different problems.

As can be seen in Figure 1, the scenarios of LPPM application are either online or offline. The online LPPMs operate between the users and LBS, and these can be further divided into real-time scenarios and batch scenarios.

In real-time scenarios, users who request LBS need to receive a quick response, for instance, in navigation and localization applications. The primary challenge faced by LPPMs in this context is that achieving location privacy protection is difficult owing to the limited location information that can be utilized within a short time slot. Furthermore, the protected location information is also constrained by the necessity for low latency.

In batch scenarios, users typically send data to LBS on a regular basis and then anticipate a response. Compared to real-time scenarios, these algorithms do not necessitate low latency, and they generally transmit a significant amount of data in a single batch.

In offline scenarios, LBS may transfer the collected location datasets to a server, such as a database. Unlike real-time protection, it is easier to implement location privacy protection with offline protection algorithms, as more information can be utilized. Furthermore, traditional LBS rely on a dedicated server to process location information, leading to a diverse range of research on offline LPPMs. However, with the advancement of the IoT and edge computing, there is a growing need to process information at the edge nodes, like Access Points (APs) and Base Stations (BSs) [23], making LPPMs for online scenarios increasingly crucial. In the subsequent sections of this paper, a novel online LPPM called CKA is proposed for protecting the location privacy of IoT devices in an edge computing environment.

### 2.2. Existing Privacy-Preserving Mechanisms

K-anonymity and differential privacy are widely studied and utilized techniques for location privacy protection [27]. Differential privacy, another key area, includes centralized (CDP) and local (LDP) approaches, depending on the trustworthiness of third-party data collectors.

The concept of K-anonymity was introduced by Samarati and Sweeney in 1998. With the advancement of technology, there exists a variety of K-anonymity algorithms for data protection. For instance, a heuristic K-anonymity algorithm, Datafly is discussed in [28], which summarizes the quasi-identifiers exhibiting the most disparate values [29]. Y. Zhao et al. utilize the kd-tree to segment the dataset and reconstitute it using at least K-equivalent records [30].

One of the classic applications of K-anonymity is to protect the trajectory of a node; its core concept involves confusing eavesdroppers by publishing a trajectory that includes the real information alongside K-1 fake trajectories [31]. To address the challenge of creating a similar mobility pattern, several models have been proposed, including the random walk mobility model, the random way-point mobility model, and the random direction mobility model [32].

In the random walk mobility model [33], all the basic elements of a node are random, encompassing the location, speed, and direction. Whenever the time interval reaches a predefined threshold since the last move, the node will relocate to a random position. In contrast, in the random way-point mobility model [34], the node has a random speed, direction, and even destination, allowing it to move freely. Before reaching its destination, the node will pause when the elapsed time matches a set value and then return along the same route. In the random direction mobility model [35], the node’s route is random, but it must move towards a defined area or the boundaries of the simulation area. Upon reaching the area or boundary, the node pauses for a period before selecting a new direction and continuing to another area.

Differential privacy is also a pivotal research area in privacy protection. Existing differential privacy technologies can be categorized into two main types: CDP technology and LDP technology [36]. CDP involves gathering the raw data into a data center, where differential privacy protection algorithms are then applied to the data. This approach assumes that third-party data collectors are trustworthy. Conversely, LDP is founded on the premise that third parties cannot be trusted and that all personal data are managed and protected directly by users themselves. L. Sun et al. propose a locally differential private bit vector mechanism with a distance-aware property in the anonymous space [37].

In conclusion, while research on LPPMs encompasses diverse aspects, it is frequently constrained by factors such as location and the number of users, as current studies predominantly rely on either real nodes or virtual nodes, with a primary focus on the remote server. This research, however, emphasizes protecting location privacy by generating a variable number of virtual nodes within zones after implementing reasonable clustering. This approach aims to protect location privacy in IoT environments with edge computing capabilities and alleviates the issue of heightened exposure risk stemming from a dense concentration of nodes within a specific region.

## 3. System Model

**Figure 2:**
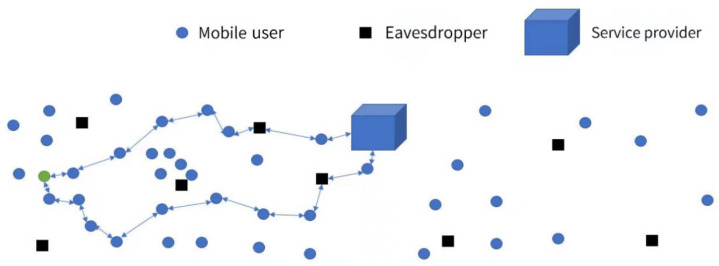
System model of service based on locations in the IoT.

As shown in Figure 2, the IoT comprises mobile users and service providers, where all the circles represent legitimate users. However, some “users” act as eavesdroppers, posing a threat to the privacy of others. The architecture of CKA is peer-to-peer, and the CKA process incorporates generalization (i.e., K-anonymity), dummies (i.e., generating virtual nodes), and protocols (i.e., determining whether a user acts as a head node). It is important to mention that the nodes considered in this paper are mobile, and a brief description of each type of device is as follows:

Mobile user: A mobile user is a type of legitimate mobile device (e.g., robots, WSN nodes, etc.). When a mobile user acts as the head node, such as thee green circle in Figure 2, it broadcasts to the surrounding region without revealing its location, declaring that it will assist users in sending LBS requests. If necessary, a ticket (public or temporary key) is also included in the broadcast. When a user who requires LBS immediately receives the broadcast from the head node, they respond with their location and related LBS requests (encrypted or not). The head node then gathers the locations and corresponding requests from its neighbors and sends them to the service provider, even if the number of responses is less than *K*. This serves as the foundation upon which CKA can be implemented.

Service (LBS) provider: The service provider can be any device that can meet the needs of users with the capability of edge computing. When the service provider receives requests from users, it processes the requests according to their related locations and then responds to the user.

Eavesdropper: An eavesdropper is a device that listens on the communication channel between users and the service provider and impersonates a real user. When an eavesdropper receives broadcasts from head nodes, it responds to its location as a real user.

## 4. Clustering K-Anonymity Algorithm

For protecting the location privacy of users during the communication process of the IoT with edge computing, traditional K-anonymity algorithms can utilize the locations of real nodes (real locations) around some users or the location of virtual (dummy) nodes (virtual locations) to achieve K-anonymity. However, the eavesdropper can narrow down the region where the user is located according to the real locations, and the virtual locations will limit communication efficiency, where more requests need to be sent.

Different from traditional K-anonymity algorithms, the CKA introduces the idea of clustering to confuse the eavesdropper, where the real locations are uniform in a selected region. Moreover, the number of virtual locations is optional so that the communication efficiency is acceptable for users, while the security of location privacy is improved when compared with the real-location-only K-anonymity. To facilitate understanding of the ideas of CKA, we assume that each location, including the virtual location, is sent with its related request. In the rest of the paper, *K* refers to the degree of anonymity, which signifies the number of locations in each request to the service provider. Similar to traditional K-anonymity algorithms, the larger *K* is, the stronger the privacy protection capability becomes. *N* is the number of clusters, which represents the number of zones divided from the selected region.

The principle of CKA is as follows:

Phase 1: The initiating node will monitor the broadcasts from the head node to determine if it should be a normal node, where the head node is the user that sends the *K* locations to the service provider, and the others are normal nodes. It can be explained according to Equation (1),

(1) $$l_{\tau }=\left\{\begin{array}{cc} 1, & \mathrm{if}\;b_{\tau }>0\\ 0, & \mathrm{otherwise}. \end{array}\right.$$

where $b_{\tau }$ indicates the number of broadcasts from head nodes during the time interval τ, and, therefore, if there exists any broadcast, $b_{\tau }$ > 0. $l_{\tau }$ is an indicator function; the user needs to select one head node to send its response if $l_{\tau }$ = 1. If there is no broadcast, i.e., $b_{\tau }$ = 0, then $l_{\tau }$ = 0, the user will send no response. After that, it acts as the head node to initiate and broadcast a notice to collect locations from neighbor nodes and then turns to Phase 2.

Phase 2 (head node only):The region surrounding the head node is divided into *N* zones and nodes in the same zone form a cluster. It is obvious that at least one location, real or virtual, from each cluster should be included in the request to the service provider. Thus, the location information within the requests can be uniformly distributed across the region.

Theoretically, the number of real nodes in each request should not exceed *K*; however, the inclusion of virtual nodes is guaranteed only if K<N, and this scenario degrades CKA to traditional K-anonymity. To ensure that CKA operates effectively, CKA maintains the number of selected real nodes to no more than <γK/N>, where “<>” denotes “round”, and 0<γ≤N is a weight parameter. The utilization of γ makes the security and the communication efficiency of CKA controllable—in other words, the security of location privacy and the communication efficiency can be adjusted by *N* and γ.

To balance the number of nodes of each cluster in the request sent to the service provider, and to expand the possible region of real nodes that the eavesdroppers identify according to the locations in K-anonymity, the number of nodes in each cluster should be as close as possible. If the number of real locations in cluster *i* is less than ⌊K/N⌋, the head node creates $f_{\tau }\left(K,N,i\right)$ virtual nodes during time interval τ to make sure the locations of each cluster are as similar as possible, as shown in Equation (2),

(2) $$f_{\tau }\left(K,N,i\right)=\left\{\begin{array}{cc} r_{\tau }\left(K,N,i\right), & \mathrm{if}\;a_{\tau }\left(i\right)\leq T\left(K,N\right)\\ 0, & \mathrm{otherwise}. \end{array}\right.$$

where $a_{\tau }\left(i\right)$ is the number of received responses in cluster *i*, and T(K,N) is a threshold, as shown in Equation (3).

(3) T(K,N)=⌊K/N⌋−1

During the time interval τ, if $a_{\tau }\left(i\right)$ is less than ⌊K/N⌋, $r_{\tau }\left(K,N,i\right)$ virtual nodes are generated randomly in cluster *i*, as shown in Equation (4). It should be noted that the head node is assumed to send reports itself; so, no more than T(K,N) real nodes in the zone of the head node can be selected to achieve the clustering K-anonymity.

(4) $$r_{\tau }\left(K,N,i\right)=\left\lfloor K/N\right\rfloor -a_{\tau }\left(i\right)$$

If the number of receiving reports $a_{\tau }\left(i\right)>T\left(K,N\right)$, then ⌊K/N⌋ nodes are randomly selected among them. It should be noted that all the operations mentioned above are subject to the condition that the selected real nodes do not exceed <γK/N>, or the head node can only generate virtual nodes, even if $a_{\tau }\left(i\right)\leq T\left(K,N\right)$.

The probability, P(K,N), that the attacker infers the real location is shown in Equation (5).

(5) P(K,N)=<γK/N>/K

Therefore,

(6) P(K,N)∝1/N

which means that the larger *N* (the more clusters), the higher the level of location privacy security.

If N⌊K/N⌋<K, the head node should generate the remaining K−N⌊K/N⌋ virtual nodes in different clusters (each cluster accepts at most one remaining virtual node).

**Figure 3:**
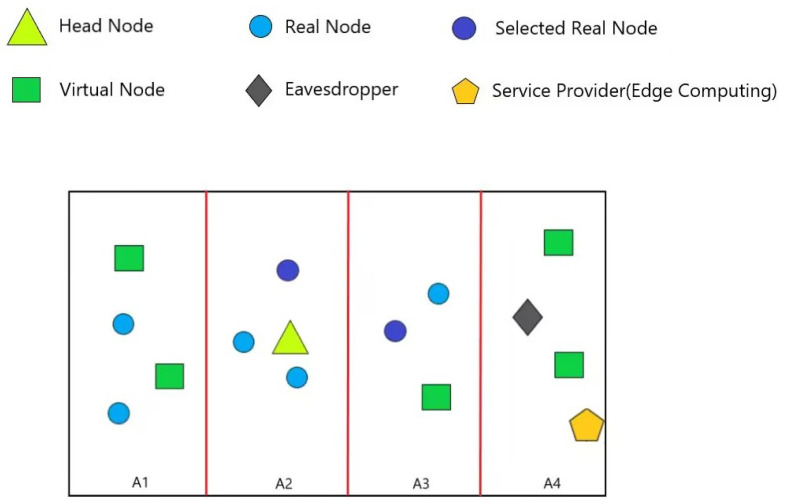
Region division mode of nodes I.

For instance, as shown in Figure 3, the region is randomly divided into four zones, which means that *N* is 4. Assuming that *K* is 8, γ=1.5, then T(K,N)=1. The head node selects A2 to make its cluster, and it needs to compare the number of responses $a_{\tau }\left(i\right)$ and the threshold 1. Fortunately, if $a_{\tau }\left(A2\right)>T\left(K,N\right)$, then the algorithm needs to select itself and another real node in A2 to achieve clustering K-anonymity. Moreover, if $a_{\tau }\left(A1\right)$ = 0, then two virtual nodes are created in A1. Similarly, two virtual nodes are created in A4. Finally, if $a_{\tau }\left(A3\right)$ = 1, then one virtual node is created in A3.

The division of the region is designed based on the application environment. One division mode involves ensuring that all of the divided zones are covered by the communication range of the head node. The selected region is expanded according to the rule that the size of each zone should be as similar as possible. After expansion, the zone where the head node is located can be positioned either at the center or the edge of the expanded region. In such a scenario, the communication range of the head node can be varied to prevent the zone in which it is located from being identified by an eavesdropper. Therefore, when *K* is large enough, the information entropy of the zone where the node is located is maximized, reaching logNbit/zone, and the uncertainty of the eavesdropper about the zone in which the actual node is located is also maximized.

**Figure 4:**
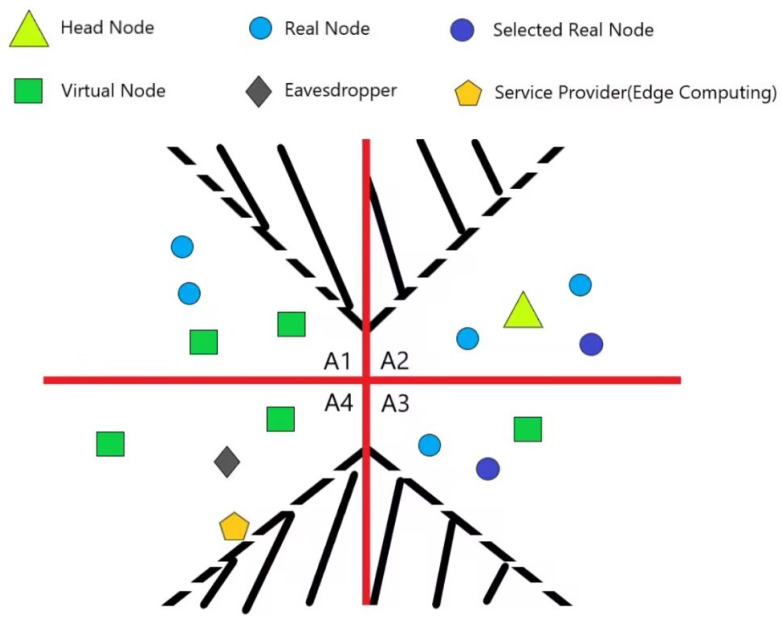
Region division mode of nodes II.

Figure 4 is another division mode for the region, where the size of each zone is unlimited, and the zone has a part that is included by the communication range of the head node. This kind of division mode is suitable for VANET in which the mobility of nodes is high.

It should be noted that the virtual nodes can be generated in any zone; therefore, the combination of the selected real nodes and the virtual nodes can make location distribution more scattered and natural. This also makes it more difficult for the eavesdropper to find the real locations.

Phase 3 (head node only): The head node broadcasts a confirmation with all locations, including the virtual locations, and then sends the request with *K* locations to the service provider. The broadcast can make sure that the selected real node is normal.

If a normal node sends its request without receiving confirmation from the head node after a while, it will repeat Phase 1. It should be noted that if the head node broadcasts the request to the service provider, then the process of confirmation is not necessary.

After executing CKA, the service provider receives a request with locations. It then responds to the request with the corresponding services, respectively. Finally, the users can obtain their services based on the locations provided in the response.

The pseudo-code about the algorithm is shown in Algorithm 1.

CKA aims to protect the location privacy of nodes during the LBS process. Therefore, it is necessary to evaluate the performance of both privacy protection and data transmission, which will be analyzed in the next section.
**Algorithm 1** Pseudo-code of CKA**Input:** K,N,γ,τ; **Output:** responses (normal node), confirmation, and service request (head node);
  1:**if** $l_{\tau }=0$ **then**  2:    The node should be a head node according to Equation (1);  3:    Broadcast a notice;  4:    $S_{\tau }$ is the number of selected real nodes;  5:    **for**  1≤i≤N **do**  6:        **if** $S_{\tau }<<\gamma K/N>$ **then**  7:           **if** $a_{\tau }\left(i\right)\leq T\left(K,N\right)$ **then**  8:               Generate $f_{\tau }\left(K,N,i\right)$ virtual nodes in zone *i* according to Equation (2);  9:           **else**10:               Select min(⌊K/N⌋, <γK/N>-$S_{\tau }$) real nodes in zone *i*;11:           **end if**12:        **else**13:           Generate K−N⌊K/N⌋ virtual nodes;14:        **end if**15:    **end for**16:    **if** N⌊K/N⌋<K **then**17:        Generate K−N⌊K/N⌋ virtual nodes;18:        Put each of the above virtual nodes into a unique cluster;19:    **end if**20:    Broadcast a confirmation with all locations;21:    Send the request to the service provider;22:**else**23:    The node is a normal node according to Equation (1);24:    Select one of the head nodes to send a response;25:**end if**


## 5. Simulation

In this section, the performance of the proposed CKA is evaluated using MATLAB 2018b on a computer equipped with an Intel Core i7 processor and 16 GB of RAM. The simulations are conducted in a 100 m × 100 m region, which could represent a smart factory or an airport with numerous robots or AGVs [38,39]. Each result presented is the average of 100,000 simulations, where each node randomly initiates an LBS request or broadcasts its location information in each time slot. The parameter γ is set to 1 to meet the normal requirements of security and communication efficiency. The number of legal users and eavesdroppers varies according to different simulation scenarios. The division mode is not specified here, as all zones are assumed under the coverage of the head node in this section, meaning that the affiliation of nodes and zones, rather than the shape of zones, will affect the results in this section.

To evaluate the proposed algorithm fairly, it is assumed that for K-anonymity algorithms, all the requests or responses from normal nodes to the head node, which will send the LBS request with the information of *K* locations, are considered secure. It should be noted that under this assumption, short-distance communications between normal nodes and their head node can be secured without encryption, which does not significantly increase the overhead of computation and communication [40]. The location privacy of the communication between the head node and the service provider (edge device) is protected by K-anonymity algorithms.

Eavesdroppers will respond to the head node randomly, just like the normal nodes, in order to prevent identification by legal users or the service provider. Once the eavesdroppers receive a response from the head node, the real locations can be estimated with some probability. It should be noted that the “direct transmission” in the simulation refers to a scenario where the location-based server is not colluded with or compromised by the eavesdropper (since, once colluded with or compromised, the “direct transmission” cannot avoid eavesdropping).

On the other hand, the traditional K-anonymity algorithm used in the simulation does not require a third-party server. Only a real node can be included in the requests of traditional K-anonymity, and it is assumed that the eavesdropper is aware of the algorithm. Finally, the CKA assumes that the eavesdropper knows the algorithm and can collude with the service provider (edge device).

**Figure 5:**
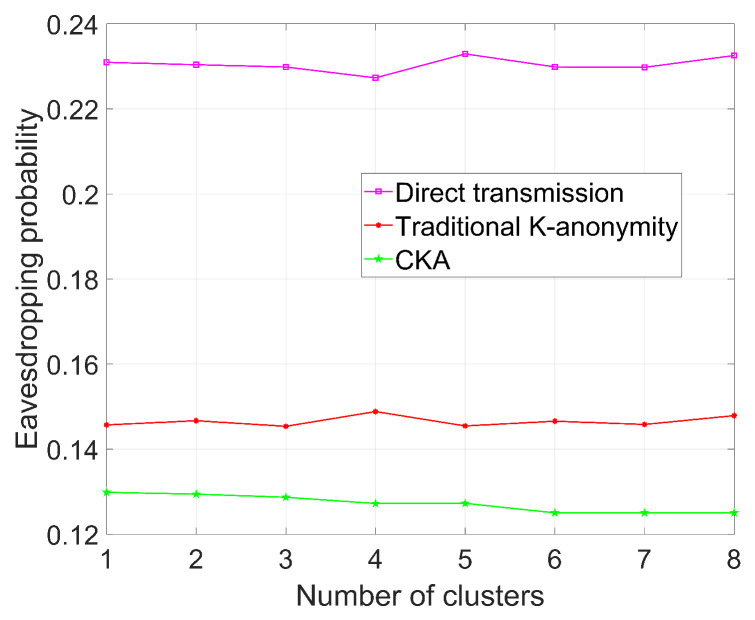
Eavesdropping probability with different number of clusters.

**Figure 6:**
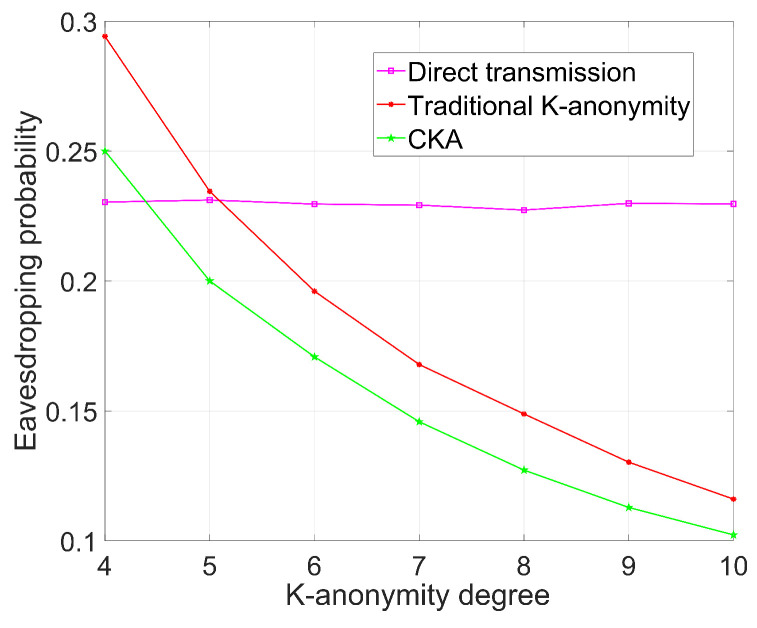
Eavesdropping probability with different K-anonymity degree.

**Figure 7:**
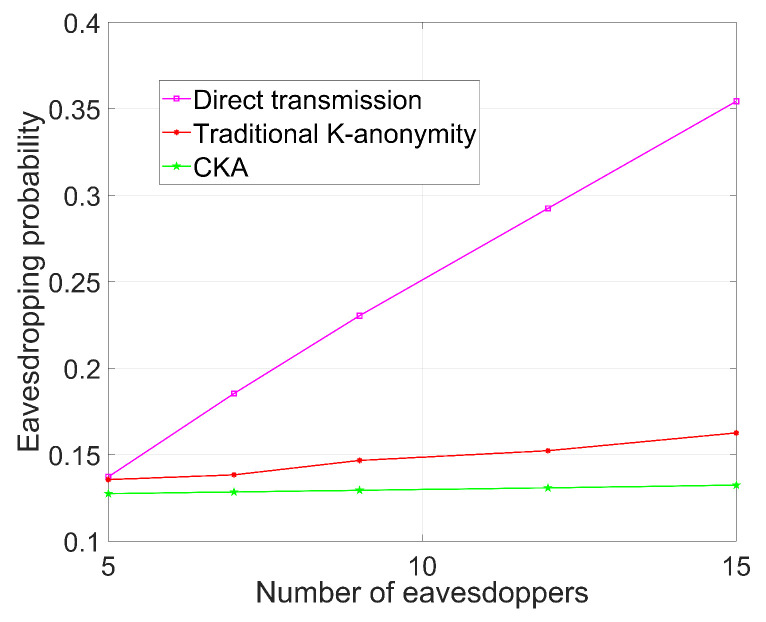
Eavesdropping probability with different number of eavesdroppers.

**Figure 8:**
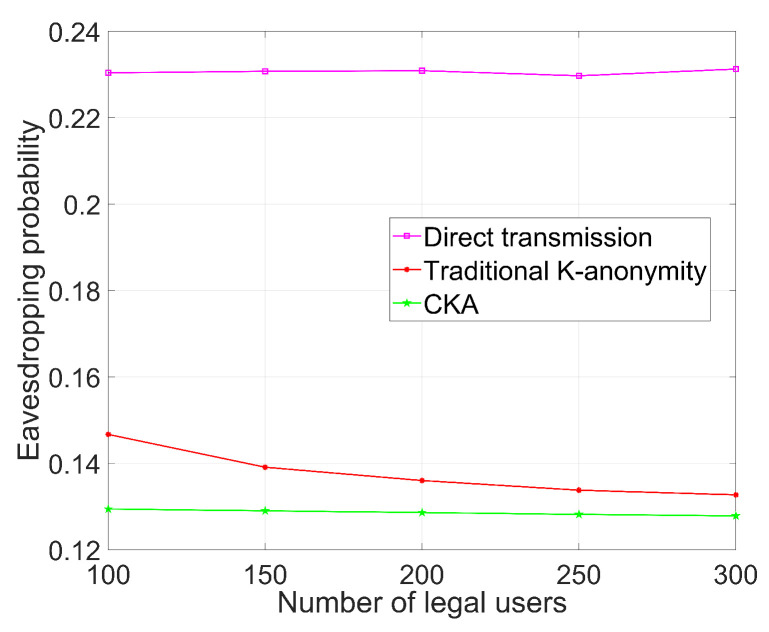
Eavesdropping probability with different number of legal users.

Figure 5, Figure 6, Figure 7 and Figure 8 demonstrate the change in eavesdropping probability (the possibility of being eavesdropped) with different variables, where *N* is set to 4 except that in Figure 5. The numbers of legal users and eavesdroppers are set to 100 and 9, respectively, in the simulations presented in Figure 5 and Figure 6.

### 5.1. The Influence of Different Number of Clusters on Security

As depicted in Figure 5, where K=8, the eavesdropping probability of direct transmission fluctuates around 0.23, and the eavesdropping probability of the traditional K-anonymity algorithm fluctuates around 0.15. As anticipated, the eavesdropping probability of CKA decreases as the number of clusters increases, and it is evident that the eavesdropping probability of CKA is lower than that of the traditional K-anonymity algorithm. According to Equation (3), if *N* increases, T(K,N) will decrease, resulting in a decrease in the number of real nodes in a CKA request, which, in turn, leads to a lower eavesdropping probability. Since the experiment employs a rounding operation for T(K,N), it can be observed that the eavesdropping probability of CKA remains unchanged when the number of clusters is 4 and 5, where T(K,N)=1. Consequently, the eavesdropping probability does not vary in these cases.

In Figure 5, it is clear that the eavesdropping probability of CKA is lower than the traditional K-anonymity algorithm under the same parameters. Furthermore, the eavesdropping probability of the traditional algorithm is lower than that of direct transmission. Since no clusters exist in direct transmission and the traditional K-anonymity algorithm, the results merely fluctuate. However, for CKA, as the number of clusters increases, it becomes more difficult for eavesdroppers to pinpoint users’ precise locations due to the increased number of virtual locations.

### 5.2. The Influence of Different Degrees of K-Anonymity on Security

As depicted in Figure 6, where N=4, the eavesdropping probability of direct transmission oscillates by approximately 0.23. Conversely, the eavesdropping probabilities of traditional K-anonymity and CKA diminish as the value of *K* rises. Notably, CKA exhibits a lower eavesdropping probability than the traditional K-anonymity algorithm at an equivalent *K* value due to its utilization of virtual locations, whereas traditional K-anonymity comprises both legitimate users and eavesdroppers.

Specifically, when K=4, the traditional K-anonymity method possesses an eavesdropping probability exceeding 0.25. This heightened probability stems from the collusion between the service provider and the eavesdropper, where the initial eavesdropping probability is 1/4. Additionally, since the request may encompass an eavesdropper, the eavesdropping probability surpasses 0.25.

In contrast, an eavesdropping probability of 0.25 is maintained when K=4. This is because T(K,N) equals 0 in such a scenario, indicating that the request comprises solely a single real node. Furthermore, the eavesdropping probability of direct transmission is contingent upon the count of eavesdropping nodes present. It also shows that *K* must be chosen appropriately to achieve the desired privacy protection. An inadequate *K* value may render the system vulnerable to eavesdropping, while an excessively high *K* may compromise system efficiency and usability.

### 5.3. The Influence of Different Number of Eavesdroppers on Security

In Figure 7, the parameters are the same as in the previous simulations, except that K=8 and N=2. It should be noted that a set of *N* results in a relative large T(K,N), which implies a smaller number of virtual nodes, i.e., the security of CKA can be improved by increasing *N*. It is obvious that all three algorithms’ performance degrades with an increasing number of eavesdroppers, but the eavesdropping probability of CKA is not as sensitive to the number of eavesdropping nodes as the K-anonymity algorithm, and certainly, direct transmission is the most sensitive. Specifically, regarding the eavesdropping probability, the increasing rate of CKA is smaller than that of the traditional K-anonymity algorithm, while the increasing rate of direct transmission is much higher than the other two. Furthermore, the eavesdropping probability of CKA is always the lowest. Direct transmission offers no protection against eavesdroppers, so the eavesdropping probability increases linearly with the number of eavesdroppers. However, compared to direct transmission, K-anonymity algorithms, especially CKA, can isolate many eavesdroppers outside the selected region. Additionally, the mechanism of creating virtual nodes can conceal users’ information, demonstrating that the eavesdropping probability of CKA is much more stable than that of the traditional algorithm.

### 5.4. The Influence of Different Number of Legal Users on Security

In Figure 8, the parameters are the same as those in Figure 7 except that the number of eavesdroppers is 9. The eavesdropping probability of direct transmission does not change with the number of legal users; it continues to fluctuate around 0.23. The eavesdropping probability of the traditional K-anonymity algorithm keeps decreasing as the number of legal users increases, while CKA also decreases slightly with an increasing number of legal users. However, it is evident that the decline in CKA is not as fast as that of traditional K-anonymity. Given that CKA is more resistant to changes in the number of eavesdropping nodes, as discussed above, we can conclude that CKA is more stable. The eavesdropping probability of CKA does not fluctuate much, even for relatively drastic environmental changes, which enhances the flexibility of CKA. Additionally, the eavesdropping probability of CKA is always lower than that of the traditional algorithms. For direct transmission, even if there are more legal users, it is still vulnerable, so the eavesdropping probability remains high. For K-anonymity algorithms, as the number of legal users increases, the exact information about *K* users becomes more vague. Consequently, it becomes harder for an eavesdropper to locate the real user, resulting in a lower eavesdropping probability than that of direct transmission. The result shown in Figure 8 indicates that, due to the clustering mechanism, even with a limited number of legal users, CKA performs excellently and is, therefore, effective for different user densities.

### 5.5. Transmission Failure

**Figure 9:**
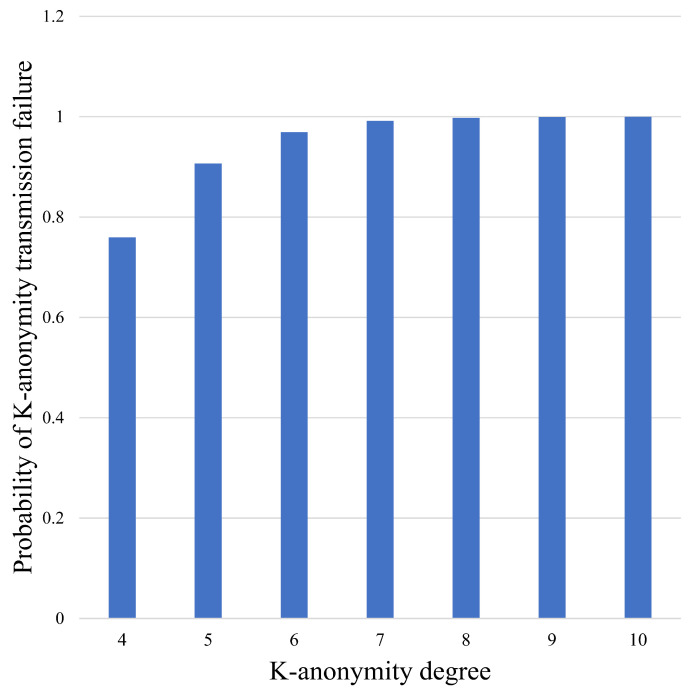
Transmission failure probability of traditional K-anonymity with different K-anonymity degree.

**Figure 10:**
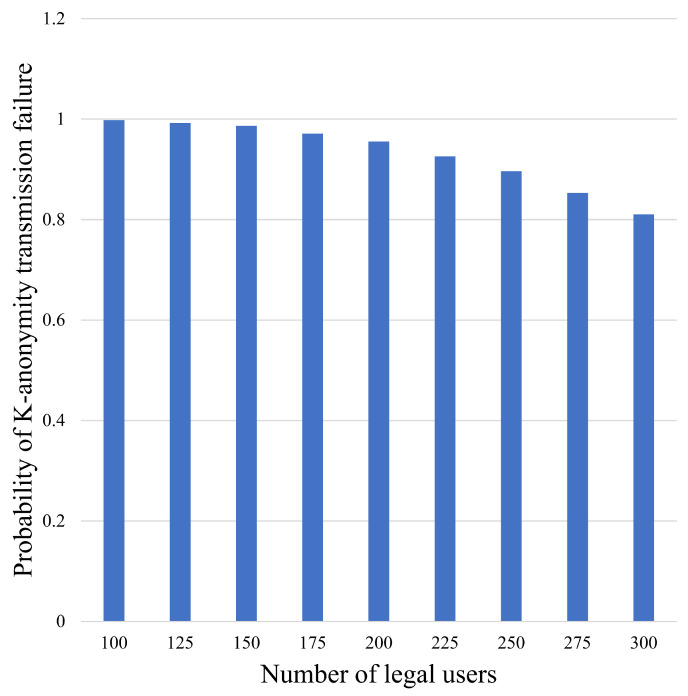
Transmission failure probability of traditional K-anonymity with different number of legal users.

Figure 9 and Figure 10 demonstrate the change in the transmission failure probability of traditional K-anonymity, which refers to the probability of re-initiating when the specified user receives less than *K* information from neighbor nodes.

As shown in Figure 9, under the above experimental parameters, the transmission failure probability of the K-anonymity algorithm increases with increase in the K-anonymity degree. When K=5, the failure probability already reaches 0.91, while the eavesdropping probability of traditional K-anonymity is nearly equal to that of direct transmission at this degree, as shown in Figure 6. When K=10, the eavesdropping probability is already close to 1; so, at this degree, the traditional K-anonymity algorithm cannot work well. On the other hand, due to the mechanism of virtual nodes, the CKA will always succeed in initiating the request, and the eavesdropping probability decreases with increase in *K*.

As shown in Figure 10, the transmission failure probability of the traditional K-anonymity algorithm decreases as the number of legal users increases and the effect is relatively impressive. However, the failure probability still reaches 0.81 in the case of 300 legal users. As the number of legal users increases, there is more users’ information that can be selected, so that the probability of finding *K* users’ information is increased, and then the transmission failure probability of the traditional K-anonymity algorithm is decreased. The traditional K-anonymity algorithm can succeed in initiating a request once there are enough users; however, the requirement is strict. Because of the utilization of virtual nodes, CKA can always transmit data successfully, and the possibility of unsuccessful transmission of CKA is 0.

### 5.6. Comparison

In our simulations, the comparison with virtual-location-only K-anonymity was omitted because it is a special case of CKA. If there are no users or any eavesdropping nodes in the selected region, the proposed algorithm degrades into a virtual-location-only K-anonymity algorithm, and this ensures that CKA initiates a request successfully every time.

The simulation results clearly demonstrate that CKA can significantly reduce the eavesdropping probability in most cases, compared to direct transmission and traditional K-anonymity. Furthermore, the privacy protection performance of CKA can be tailored by adjusting the number of clusters and the value of *K*. Specifically, increasing *K* or the number of clusters *N* leads to a notable enhancement in privacy protection, albeit at the cost of increased communication overhead, as this necessitates the inclusion of more virtual locations in the request. Consequently, it is necessary to carefully select the parameters based on the specific requirements for privacy protection and communication overhead when utilizing CKA. Moreover, CKA exhibits a more stable privacy protection performance than traditional K-anonymity across varying numbers of eavesdroppers or legal users, suggesting its greater adaptability to diverse environments.

The simulation results also indicate that traditional K-anonymity may fail to transmit a request due to the scarcity of normal nodes, whereas the transmission of requests using CKA is consistently successful. This suggests that, compared to CKA, traditional K-anonymity needs more communications to establish a K-anonymity request if the node density is not high enough. On the other hand, virtual-location-only K-anonymity can indeed transmit requests successfully, but in most cases, the communication overhead is higher than that of CKA, stemming from the fact that only one real node is involved in each request. Consequently, CKA can balance the performance of privacy protection and communication overhead by adjusting its parameters, ensuring that requests are always transmitted successfully.

In CKA, to enhance efficiency, once the head node receives *K* real locations, including its own location, it will disregard any subsequent responses from normal nodes. In such a scenario, the head node must determine whether to select each location individually, requiring at most *K* operations. Additionally, the head node needs to generate, at most, K−1 virtual locations, resulting in a computational complexity of O(2K), which is slightly higher than that of traditional K-anonymity. The larger the value of *K*, the higher the computational overhead.

As a conclusion, the performance comparison of different methods is listed in Table 1.

CKA can not only protect location privacy from traditional attacks but can also mitigate the impact of narrow-region attacks, whereas traditional K-anonymity offers no such assistance. Furthermore, virtual locations can diminish communication efficiency, as requests and responses related to them are unproductive for real nodes. Consequently, in the subsequent section, the performance of CKA in defending against narrow-region attacks will be evaluated through the design of an evaluation function, and the trade-off between certain parameters and the communication efficiency (hereafter referred to as “data efficiency” to avoid confusion) of CKA will also be assessed.

## 6. Discussion

### 6.1. Security Analysis with Regards to the Region

One of the crucial features of CKA is that it can confuse the eavesdroppers not only with regards to locations but also with regards to the region. In this subsection, we utilize the K-function to analyze the performance of CKA in terms of region confusion. The K-function serves as a statistical measure to quantify the degree of dispersion in the distribution of nodes, with a lower K-function value indicating a more uniform distribution of nodes. Within a given region, a more uniform distribution of nodes makes it harder for attackers to narrow down the region where their target may be located. Therefore, assessing the uniformity of locations within K-anonymity requests using the K-function can reflect the security of CKA in terms of region confusion. It is worth noting that the division mode in this subsection is the same as that in Figure 3.

The process of analyzing begins by recording the locations of *K* nodes and calculating the distances between them. Subsequently, for each node *p*, the minimum distance $d_{min}^{\left(p\right)}$ to the other K−1 nodes is recorded, say *q*, as shown in Equation (7).

(7) $$d_{min}^{\left(p\right)}=\underset{q\in [1,K],q\neq p}{min}|X_{p}-X_{q}|$$

Among them, $X_{p}$ and $X_{q}$ are the coordinates of nodes *p* and *q*, respectively. Then, the value of the K-function, $D_{K}$, can be obtained by calculating the variance of these minimum distances, as shown in Equation (8).

(8) $$D_{K}=\frac{1}{K}\sum_{P=1}^{K}{\left(d_{min}^{\left(p\right)}\right)}^{2}-{\left(\frac{1}{K}\sum_{P=1}^{K}d_{min}^{\left(p\right)}\right)}^{2}$$

Therefore, the K-function inherently computes the variance of the minimum distances among nodes, and a larger variance may suggest an increased vulnerability to the eavesdropping of nodes’ regions. Additionally, uniform distribution cannot ensure that the region occupied by the *K* nodes is sufficiently large. To address the above problem, we propose a comprehensive evaluation function to assess the security of location privacy under different clustering conditions. Specifically, to represent the size of the region where *K* nodes are distributed, the mean of the maximum distances, $d_{\max}^{\left(p\right)}$, between each node and the remaining K−1 nodes is calculated, and this mean is then weighted with $D_{K}$ to yield the final evaluation result $A_{K}$, as shown in Equation (9).

(9) $$A_{K}=\beta \frac{1}{K}\sum_{P=1}^{K}d_{max}^{\left(p\right)}-D_{K}$$

where β is the adjustment factor.

(10) $$d_{max}^{\left(p\right)}=\underset{q\in [1,K],q\neq p}{max}|X_{p}-X_{q}|$$

As shown in Equation (9), when the node density is high, the mean value of $d_{max}^{\left(p\right)}$ is more influenced by clustering, while the node distribution itself has less influence on it, and in such a situation, β can be set to a smaller value. When the node density is low, the node distribution has a greater influence on the mean value of $d_{max}^{\left(p\right)}$; therefore, β can be set to a larger value to prevent the impact of the nodes’ uniformity on the evaluation function from being too small.

It is obvious that as long as the appropriate adjustment factor is set, the larger the $A_{K}$ is, the greater the nodes’ dispersion is and the higher the uniformity is. A larger $A_{K}$ makes it more difficult for eavesdroppers to narrow down the possible region of their attack targets, and effectively ensures the privacy and security of the location of each node. Moreover, $A_{k}$ provides a more intuitive insight into the capability of CKA to safeguard user information under various conditions.

A denser distribution of nodes within the same region indicates a higher likelihood for attackers to achieve their target, e.g., by limiting the region where the real user is located. Therefore, for the CKA algorithm, a lower node density implies a smaller number of real nodes, making it more difficult for real node information to be intercepted.

In the following analysis, each result is the average of 100,000 iterations, the legitimate user and the eavesdroppers are 540 and 90, and the adjustment factor β is determined through testing to be an appropriate value before being incorporated into the experiments.

**Figure 11:**
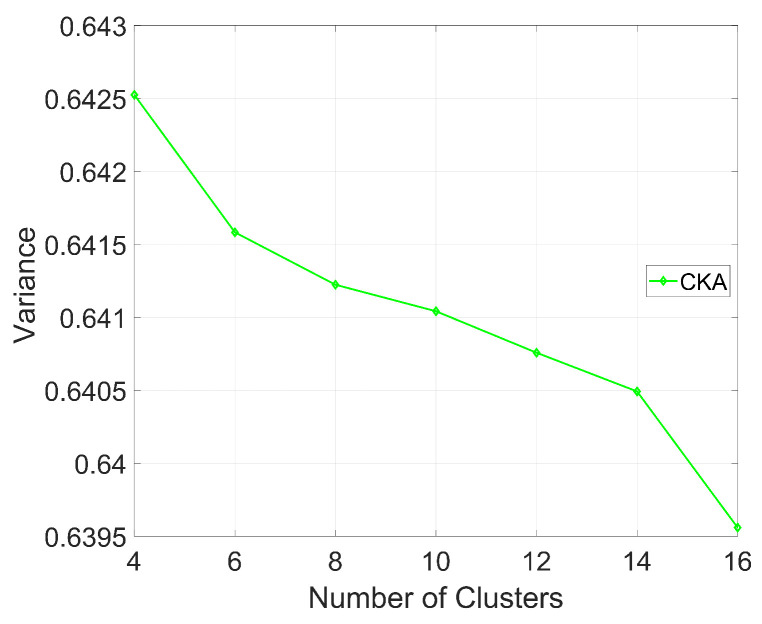
The variation in the variance of the minimum distance between nodes as the number of clusters changes.

Figure 11 shows the value of the K-function with different clusters, where K=16, respectively. The results show that as the number of clusters increases, the value of the K-function gradually decreases. With an increasing number of clusters, nodes in the network are more evenly distributed among various clusters, resulting in a more compact spatial distribution of nodes within each cluster. This compact distribution leads directly to a reduction in the variance of the minimum distance between nodes. We can conclude that with an increasing number of clusters, the robustness of CKA is improved by blurring the region of users.

**Figure 12:**
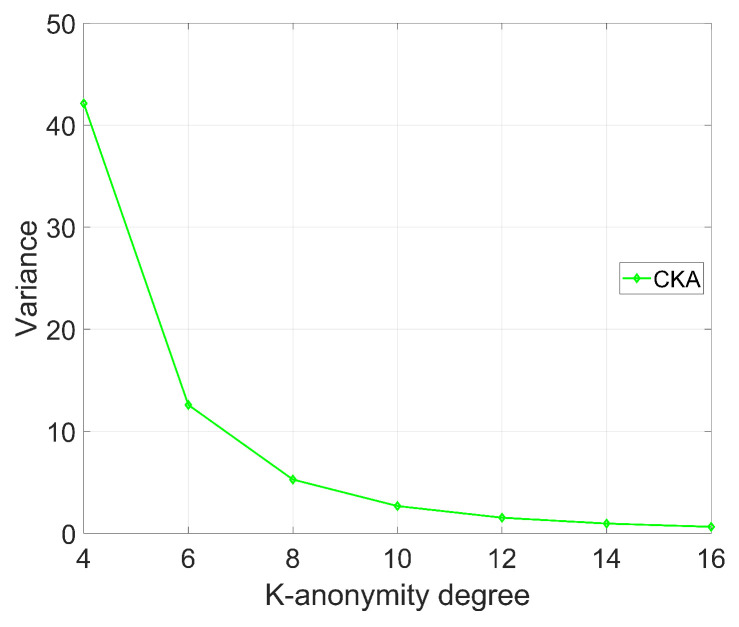
The variation in the variance of the minimum distance between nodes as the K-anonymity degree changes.

Figure 12 shows the value of the K-function with different *K*, where the number of clusters N=4. It can be seen from Figure 12 that with increase in the anonymity level *K*, the value of the K-function exhibits a notable downward trend; this is attributed to the fact that when the anonymity requirement is raised, the number of nodes included in the requests also increases. To meet the higher anonymity criteria, the CKA algorithm selects more nodes in each cluster, and then the distances between the selected nodes become closer, resulting in a significant reduction in the variance of the node distances.

It is noteworthy that as the anonymity level *K* continues to increase, the degree of reduction in the node distance variance becomes less pronounced for the same incremental change in *K*. In fact, within a fixed region, as the number of nodes increases, the space between nodes becomes more crowded. Therefore, even if *K* is further increased, the reduction in the distances between nodes becomes relatively limited due to the lack of sufficient space for the nodes to further reduce their K-function. Furthermore, this phenomenon implies the influence of node selection strategies and region constraints on the security performance of CKA. When designing the algorithm, these factors must be taken into account to ensure that anonymity requirements are met while minimizing the variance of the minimum distances, thereby enhancing the security of CKA.

**Figure 13:**
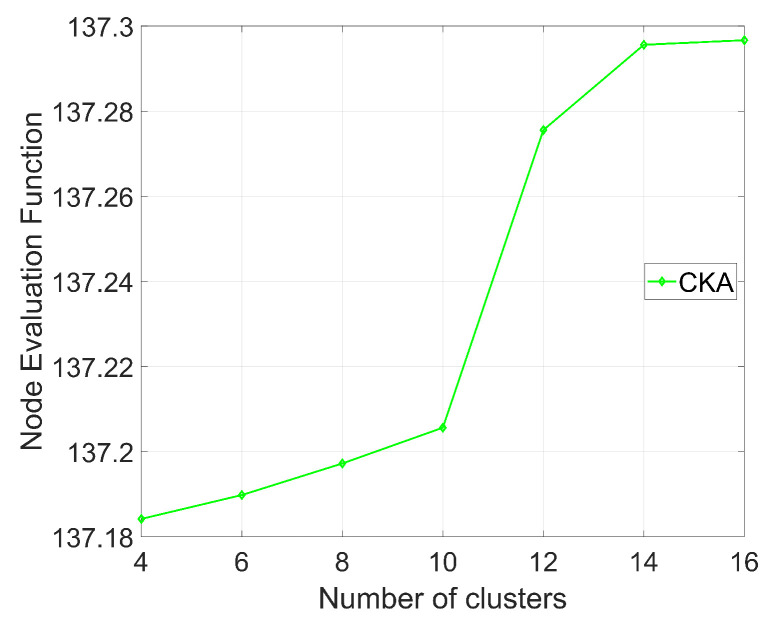
The change in the node evaluation function as the number of clusters changes.

Figure 13 shows the node evaluation function $A_{K}$ with different number of clusters, where K=16, and β=10. It can be seen from Figure 13 that as the number of clusters increases from 4 to 16, the evaluation function $A_{k}$ consistently increases because the essence of the evaluation function $A_{k}$ lies in the difference between the variance of the maximum distance and the variance of the minimum distance between nodes. When the variance of the maximum distance between nodes increases, the relationship between nodes becomes more complex and random, and, therefore, it becomes more difficult for eavesdroppers to limit the possible region of real nodes through analyzing the distance patterns. Additionally, as the variance of the minimum distance decreases, it becomes harder for eavesdroppers to identify key nodes by analyzing the distribution of nodes. Consequently, the results demonstrate that the CKA algorithm enhances the privacy protection capabilities as the number of clusters *N* increases.

**Figure 14:**
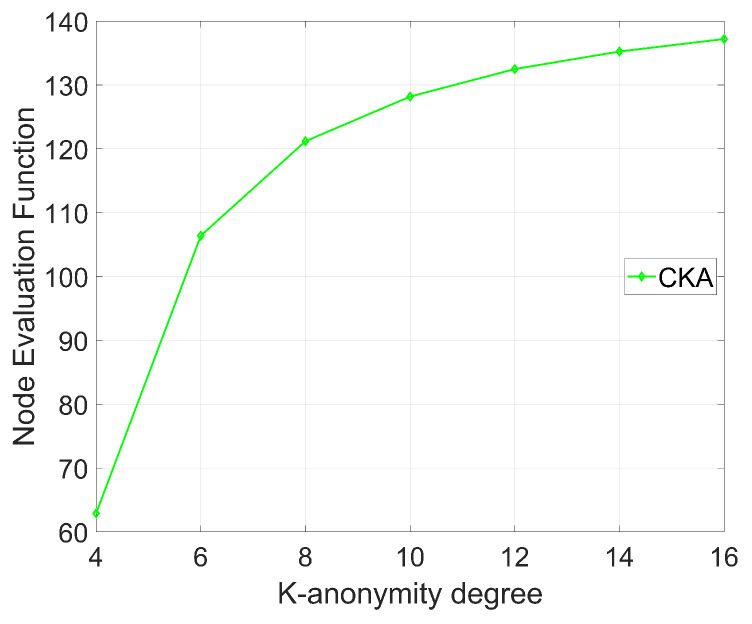
The change in the node evaluation function as the K-anonymity degree changes.

The node evaluation function $A_{k}$ with different *K* is shown in Figure 14, where N=4, and β=2. As shown in Figure 14, the evaluation function $A_{k}$ increases with augmentation of the K-anonymity degree. When K increases, the maximum distance between nodes may also enlarge, while the variance of the minimum distances gradually decreases due to increase in the number of selected nodes. Consequently, as *K* grows, the node evaluation function reflects an improvement in the algorithm’s security. However, the increasing rate of $A_{k}$ reduces with increase in *K* because the selected nodes become increasingly concentrated within clusters.

### 6.2. Application Analysis

In practical applications, to obscure the locations of the real nodes as much as possible, the density of real locations near the head node is designed to be similar to the density of the *K* locations in the selected region. Consequently, it becomes difficult for an attacker to accurately estimate the zone of real nodes based on solely on the density of locations, resulting in an uncertainty level that approaches the entropy of logNbit/zone.

If the head node receives *x* responses within the communication radius of *R*, then the node density in the current time slot is

(11) $$x/(\pi R^{2})$$

If the size of the selected region is *A*, to make it possible to include all the responses in the request from the head node to the service provider, *K* should be not less than the number of responses, as shown in Equation (12).

(12) $$K\geq Ax/(\pi R^{2})$$

Suppose that the selected region is divided into *N* zones and each response can be taken into account; then, the minimum number of nodes in each zone is

(13) $$\left\lceil Ax/\left(N\pi R^{2}\right)\right\rceil$$

so that

(14) $$K\geq N\lceil Ax/\left(N\pi R^{2}\right)\rceil$$

On the other hand, if *K* does not meet the above formula, the algorithm can still run normally, but the level of location privacy protection may be reduced because the request includes only a few or even none of the virtual locations.

CKA is designed for mobile nodes, making it difficult for eavesdroppers to discern real locations by comparing different requests, as both real and virtual node locations are variable.

However, if all nodes in the network are static, an eavesdropper can uncover real locations by comparing requests at different times, as the real locations remain constant. To mitigate this risk, each node can maintain a set of virtual node databases. When the algorithm necessitates the use of virtual nodes, it randomly selects node information from the database and incorporates it into the request packet. It is crucial to note that the virtual node database can also be updated based on previously received location information from other nodes, further obfuscating the eavesdroppers on the wireless channel.

Furthermore, since each request bears a unique sequence number, the service provider’s response does not disrupt the normal functioning of other nodes, even if their real locations are utilized. Nevertheless, if service providers collude, they can enhance the likelihood of uncovering real locations by eliminating irrelevant locations. Consequently, in practice, we must assess whether it is prudent to include unimportant real locations in the database, depending on the specific scenario. For instance, in IoT environments offering edge computing services, the likelihood of wireless channels being eavesdropped upon is significantly higher than the probability of service providers colluding. Thus, it is advisable to include unimportant real-location information in the database.

### 6.3. Data Efficiency

**Figure 15:**
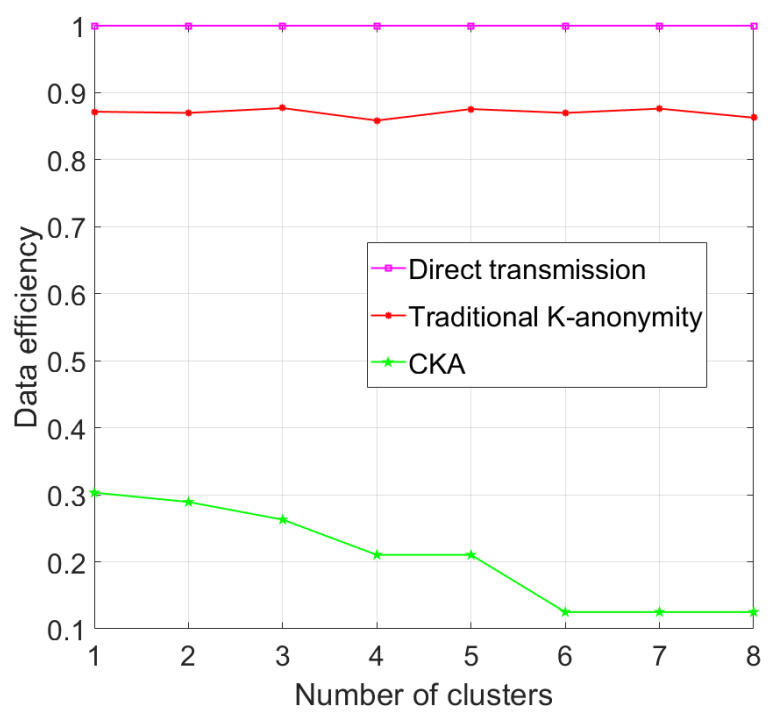
Data efficiency with different number of clusters.

**Figure 16:**
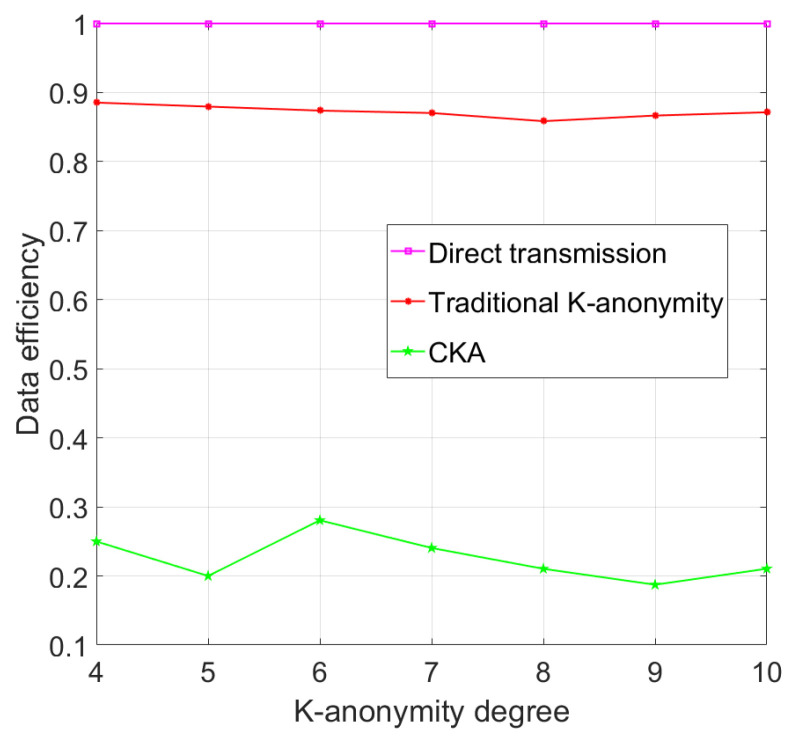
Data efficiency with different K-anonymity degree.

**Figure 17:**
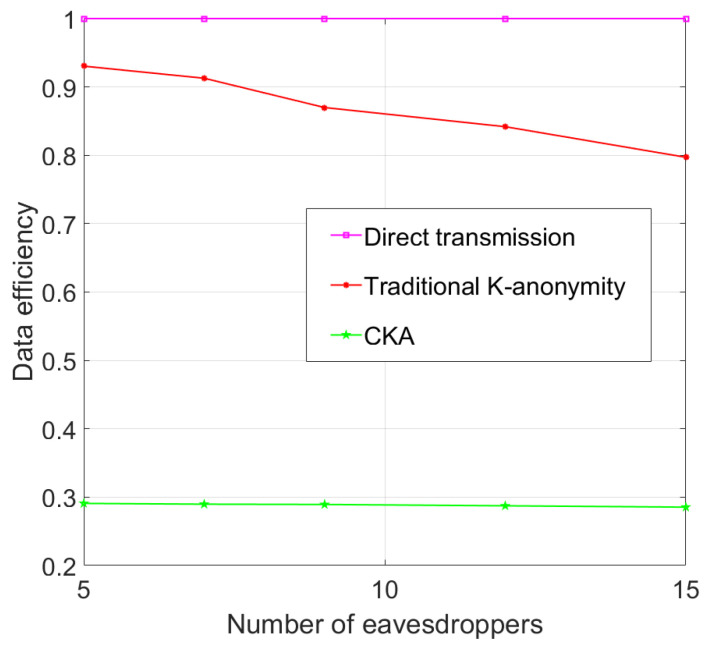
Data efficiency with different number of eavesdroppers.

**Figure 18:**
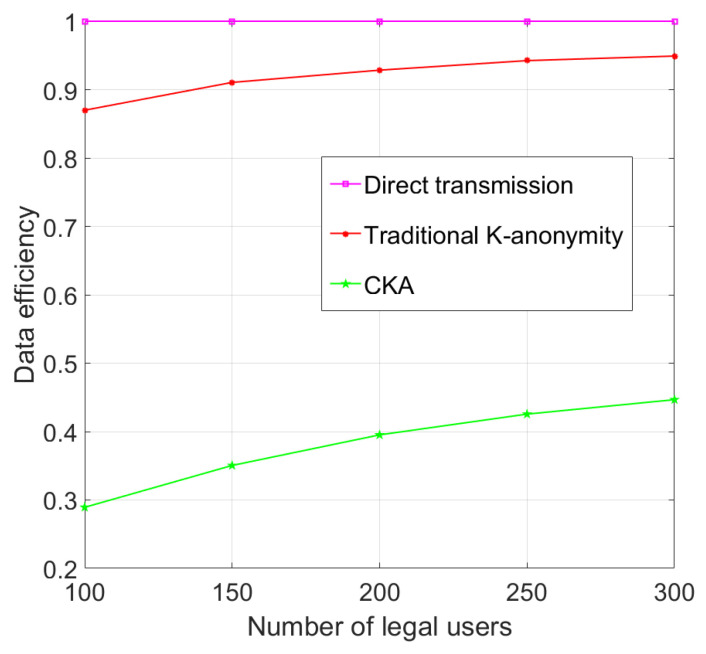
Data efficiency with different number of legal users.

Figure 15, Figure 16, Figure 17 and Figure 18 demonstrate how the data efficiency changes under different conditions. Here, data efficiency refers to the proportion of real locations within a complete request sent to the service provider. During transmission, the higher the proportion of real locations, the easier it is for information to be exposed. Therefore, it is crucial to conceal the real information. Consequently, the lower the data efficiency, the better the algorithm performs in protecting location privacy. Since the direct transmission process is directly within the eavesdropper’s attack range, its data efficiency is always 1. It should be noted that, with the parameters used in our simulation, CKA can consistently locate sufficient real nodes. Additionally, to facilitate an intuitive comparison of data efficiency, we assume that the traditional K-anonymity approach employs a virtual node rather than re-initializing data collection when transmitting the request.

As shown in Figure 15, with the same parameters in Figure 5, the data efficiency of the traditional K-anonymity algorithm fluctuates around 0.87. However, the data efficiency of CKA, as expected, decreases as the number of clusters rises. Because <γK/N> = 2, the data efficiency of CKA is stable when the number of clusters changes from 4 and 5. Similarly, the data efficiency of CKA is stable when the number of clusters changes from 6 to 8. It can be seen from Figure 15 that the data efficiency of CKA is less than that of the traditional algorithm under the same experimental parameters. However, the transmission failure probability of the traditional K-anonymity algorithm is high, which results in a longer time for the LBS process.

As shown in Figure 16, with the same parameters in Figure 6, the data efficiency of successfully executed traditional K-anonymity fluctuates around 0.87. The results show that the data efficiency of traditional K-anonymity algorithms does not vary much with change in *K* when the user density and the number of eavesdropping nodes remain unchanged. The efficiency of CKA is around 0.25, which is lower than that of the traditional algorithm, and it reveals that the efficiency of CKA varies with the degree of anonymity *K*. Because the number of clusters *N* is 4 and the degree of anonymity *K* is 4 and 5, <γK/N> = 1, CKA will generate three and four virtual nodes, respectively, and the real nodes are only one, so the efficiency of the algorithm decreases. When *K* changes from 6 to 9, <γK/N> = 2, CKA will select two nodes (legal user or eavesdropping node) to achieve anonymity, and since the number of legal users is much larger than that of eavesdroppers, the head node has a much greater chance of selecting a real node from the response nodes than selecting an eavesdropping node. Each situation of *K* will generate four, five, six, and seven virtual nodes, respectively, so the CKA shows a decrease in data efficiency when K is 6, 7, 8, or 9. When K=10, <γK/N> = 3, and the number of virtual nodes is seven, the same as that when K=9, and, therefore, the data efficiency increases.

Figure 17 shows that the data efficiency of both traditional K-anonymity and CKA decreases as the number of eavesdroppers increases, but the variation rate of CKA is not as sensitive to the eavesdropping nodes as traditional K-anonymity due to the effect of the clustering mechanism. For CKA, when the number of eavesdroppers increases, it does not mean that there will be more eavesdroppers existing in the final request, due to the clustering idea, which means the data efficiency will not change too much.

As shown in Figure 18, the efficiency of K-anonymity algorithms improves with an increase in the number of legal users, and the rate of increase for CKA is greater than that of the traditional algorithm. For the traditional K-anonymity algorithm, when the number of legal users reaches approximately 250, the data efficiency plateaus. However, for CKA, ma higher number of legal users translates to less virtual information in the final requests, thereby enhancing data efficiency. This indicates that CKA is scalable for varying user densities.

It should be noted that, while the data efficiency of CKA is often the lowest when compared to the other two schemes, the security of location privacy provided by CKA is consistently the highest. This is because CKA can safeguard location privacy even when attackers attempt to gather all real locations, whereas direct transmission and the traditional K-anonymity algorithms prove ineffective in such scenarios.

In conclusion, CKA relies on the clustering mechanism to better protect users’ privacy, and its performance improves significantly as the number of clusters increases. On the other hand, the data efficiency of CKA is influenced by the clustering mechanism; thus, the number of clusters and the value of *K* should be carefully considered based on the specific application in a real-world environment.

## 7. Conclusions

With the development of the IoT and edge computing technology, more and more LBS are being offered by edge devices, which requires applications to pay closer attention to location privacy during the communication process and within the edge devices. In this paper, a clustering-based K-anonymity algorithm, termed CKA, is proposed for protecting the location privacy of the IoT with edge computing. Unlike traditional K-anonymity algorithms, CKA does not necessitate a third-party server, making it suitable for the communication process and edge devices, even if the edge devices are colluding. Additionally, CKA can defend against narrow-region attacks, which traditional K-anonymity algorithms often fail to prevent. Furthermore, the algorithm minimizes the reliance on user density to achieve the desired performance. Moreover, simulation results demonstrate that CKA offers higher security than traditional K-anonymity algorithms and direct communication in most scenarios.

## Figures and Tables

**Table 1:** Comparison of different methods.

	P. Protection	Comp. Overhead	Comm. Overhead	T. Failure
**CKA**	High	O(2K)	Variable	0
**R. K-anonymity **	Medium	O(K)	Variable	High
**V. K-anonymity**	Medium	O(K)	High	0
**D. Transmission**	None	O(1)	Low	0

**P. = Privacy**; **Comp. = Computational**; **Comm. = Communication**; **T. = Transmission**; **R. = Real-location-only**; **V. = Virtual-location-only**; **D. = Direct**.

## Data Availability

The original contributions presented in the study are included in the article, further inquiries can be directed to the corresponding authors.
